# Exploring bacterioplankton communities and their temporal dynamics in the rearing water of a biofloc-based shrimp (*Litopenaeus vannamei*) aquaculture system

**DOI:** 10.3389/fmicb.2022.995699

**Published:** 2022-09-20

**Authors:** Su-Kyoung Kim, Jaeho Song, Meora Rajeev, Su Kyoung Kim, Ilnam Kang, In-Kwon Jang, Jang-Cheon Cho

**Affiliations:** ^1^West Sea Mariculture Research Center, National Institute of Fisheries Science, Taean, South Korea; ^2^Division of Microbiology, Honam National Institute of Biological Resources, Mokpo, South Korea; ^3^Department of Biological Sciences and Bioengineering, Inha University, Incheon, South Korea

**Keywords:** shrimp aquaculture, biofloc technique, *Litopenaeus vannamei*, bacterioplankton community, rearing water, probiotics

## Abstract

Biofloc technology (BFT) has recently gained considerable attention as a sustainable method in shrimp aquaculture. In a successful BFT system, microbial communities are considered a crucial component in their ability to both improve water quality and control microbial pathogens. Yet, bacterioplankton diversity in rearing water and how bacterioplankton community composition changes with shrimp growth are rarely documented. In this study, the Pacific white shrimp, *Litopenaeus vannamei* was cultivated in a greenhouse-enclosed BFT system. Rearing water samples were collected on a weekly basis for 5 months (152 days) and water quality variables such as physicochemical parameters and inorganic nutrients were monitored. In parallel, 16S rRNA gene pyrosequencing was employed to investigate the temporal patterns of rearing-water microbiota. The productivity, survival rate, and feed conversion ratio were 3.2–4.4 kg/m^3^, 74%–89%, and 1.2–1.3, respectively, representing successful super-intensive cultures. The metataxonomic results indicated a highly dynamic bacterioplankton community, with two major shifts over the culture. Members of the phylum *Planctomycetes* dominated in rearing water during the early stages, while *Actinobacteria* dominated during the middle stages, and *Chloroflexi* and *TM7* dominated during the late stages of culture. The bacterioplankton community fluctuated more in the beginning but stabilized as the culture progressed. Intriguingly, we observed that certain bacterioplankton groups dominated in a culture-stage-specific manner; these groups include *Rhodobacteraceae*, *Flavobacteriaceae*, *Actinobacteria,* and *Chloroflexi*, which either contribute to water quality regulation or possess probiotic potential. Altogether, our results indicate that an operationally successful BFT-based aquaculture system favors the growth and dynamics of specific microbial communities in rearing water. Our study expands the scientific understanding of the practical utilization of microbes in sustainable aquaculture. A thorough understanding of rearing-water microbiota and factors influencing their dynamics will help to establish effective management strategies.

## Introduction

The increasing human population necessitates a rapid expansion of aquaculture industries to meet the growing seafood demand (Fan and Li, 2019). Among all aquaculture sectors, shrimp production has been increasing steadily over the past few years and therefore, world’s total shrimp production in 2015 was 4.5 million tons, which was about four times higher than production in 2000 (Anderson et al., 2016). It is estimated that about 75% of farmed shrimp is produced in Asia, and most shrimp farms use semi-intensive aquaculture systems that allow flow-through of fresh rearing water to aquaculture ponds. Semi-intensive aquaculture systems encounter several challenges, including infectious diseases due to the exchange of rearing water with massive quantities of pathogen-uncontrolled water (Walker and Winton, 2010). Moreover, these systems discharge a large volume of nutrient-rich effluent, which may bring detrimental consequences (e.g., eutrophication) to the receiving water body (Páez-Osuna et al., 2003). To address these issues, several methods such as recirculating aquaculture systems and biofloc technology (BFT) have been adopted by aquaculture researchers. Though the former method is advantageous in terms of water exchange and productivity, the high operational and maintenance costs have hampered its practical deployment (Losordo and Hobbs, 2000; Gutierrez-Wing and Malone, 2006). Moreover, these systems have been shown to function weakly in terms of eliminating residual nutrients.

BFT, on the other hand, involves little or no water exchange and has recently gained considerable attention as a sustainable, cost-effective, and eco-friendly aquaculture method (Avnimelech, 2007). This method has been successfully introduced in shrimp farming (Crab et al., 2012). In principle, BFT uses a higher carbon-to-nitrogen (C/N) ratio to stimulate dense growth of the heterotrophic bacterial community. Because heterotrophic bacteria generate about 40 times more biomass than autotrophic nitrifying bacteria, aquaculture methods that favor their growth produce more bacterial biomass in the system (Brandão et al., 2021). These heterotrophic bacteria coalesce with dead organic matter as well as other microorganisms (e.g., microalgae, phytoplankton, and zooplankton) and develop flocculated aggregates (bioflocs; De Schryver et al., 2008). The densely grown heterotrophic bacterial populations actively uptake carbohydrates in rearing water and assimilate toxic inorganic nitrogen ions released in water (e.g., ammonia) to meet their nitrogen requirement (Ahmad et al., 2017). Hence, employing BFT offers three major advantages over other conventional aquaculture methods: (i) maintenance of rearing water quality by removing toxic nitrogenous constituents, (ii) minimizing the invasion of external pathogens and thus improving biosecurity, and (iii) bioflocs serve as natural proteinaceous feed for growing animals and thereby reduce the feed conversion ratio (FCR; Wasielesky et al., 2006). Considering these advantages, BFT-based operational systems have been extensively applied to shrimp aquaculture (Samocha et al., 2007).

*Litopenaeus vannamei* (Boone, 1931), also known as Pacific white shrimp and white-leg shrimp, are considered the most profitable species in the aquaculture industry (Zhang et al., 2019). Several characteristics such as their fast growth rate, high survival rate, wide range of salinity, and temperature adaptability make *L. vannamei* an excellent choice for aquaculture industries (Alfiansah et al., 2018). A study by Anderson et al. (2016) reported that *L. vannamei* alone contributed to more than 75% of the world’s shrimp production in 2015, which was about 4.2 million tons. Likewise, as per the report of the Fisheries and Aquaculture Department (FAO, Food Agriculture Organization of the United Nations, 2018), crustaceans represented 11.4% of the total aquaculture production, to which the *L. vannamei* alone contributed more than half. However, the aquaculture application of this species is associated with complications such as increased environmental pollution and frequent incidences of viral- and bacterial-originated diseases including white spot syndrome virus, early mortality syndrome, and acute hepatopancreatic necrosis disease (Huang et al., 2016; Flegel, 2019). To address these issues, BFT has been applied worldwide for *L. vannamei* aquaculture (Krummenauer et al., 2011; Correia et al., 2014). Previous studies by our research group observed that bioflocs have a positive influence on *L*. *vannamei* growth and upregulate the expression of several immunity-related genes (Kim et al., 2014, 2015).

Bacterioplankton communities and their biological functionalities are critical components of BFT (Zeng et al., 2017). The main motive behind characterizing the complex microbial communities is to develop a method that can maintain a stable microbial community, supporting optimal shrimp health and water quality. In fact, variations in microbial communities are thought to be closely associated with disease outbreaks in aquaculture systems (Xiong et al., 2015). Given that the microbial community determines water quality and the overall functioning of BFT, a number of studies have been conducted so far to describe the microbiota composition of various components of the BFT system. For example, Cardona et al. (2016) investigated bacterial community composition in the intestine of *Litopenaeus stylirostris* and rearing water of both clear seawater and BFT systems and revealed that bacterial communities of both systems varied considerably. Subsequently, carbon sources and the C/N ratio were demonstrated to influence the bacterial community composition of BFT (Deng et al., 2018). It was also demonstrated that the gut microbiota composition of *L. vannamei* changes dramatically during various growth stages (Kim et al., 2021), and that gut microbiota share a strong resemblance with bacterial communities of large-sized bioflocs (Huang et al., 2020). Overall, sufficient studies demonstrating variation in shrimp intestinal microbiota during various growth stages are available in the literature (Zeng et al., 2017; Xiong et al., 2018). However, studies delineating bacterioplankton community dynamics in rearing water at various culture stages, and its association with prevailing environmental variables in a BFT system, are rather scarce.

Henceforth, in the present study, we attempt to culture the shrimp *L*. *vannamei* in a greenhouse-enclosed production system, operating based on BFT. The growth pattern of *L*. *vannamei* and physiochemical properties of rearing water were monitored for five consecutive months. Concurrently, high-throughput amplicon sequencing was used to investigate how the bacterioplankton community of the rearing water changed with shrimp growth to explore the intrinsic association between culture stages, environmental variables, and microbiota composition of rearing water.

## Materials and methods

### Establishment of indoor shrimp production system and preparation of rearing water

A greenhouse-enclosed biofloc aquaculture system for shrimp production was constructed at the West Sea Mariculture Research Center, National Fisheries Research and Development Institute, Taean, South Korea (Supplementary Figures S1, S2). This prototype system had two 300 m^2^ raceways (with a water depth of 0.8 m) enclosed in a 1,000 m^2^ greenhouse structure with double-layered translucent sheets for heat insulation. The two raceways (Tank-1 and Tank-2), operating based on the BFT, were enclosed by linear low-density polyethylene sheets and had central longitudinal concrete partitions to facilitate water circulation. Polyvinyl chloride (PVC) pipes (50 mm in diameter) were placed at the bottom of the raceways to provide oxygen-rich water and connected with two 3 hp. water pumps. Another PVC pipe on the bottom had injector nozzles (20 mm in diameter) to suspend particulate matters. Each raceway was equipped with six airlifts (5 cm in size) on each side of the partition and also with a foam fractionator to remove excessive particulates. Rearing water was not exchanged during the 152 days of the trial and was heated by a heat-exchanger using groundwater. Foot basins for disinfection using sodium hypochlorite solution (10%) were placed at each entrance.

For the preparation of rearing water, incoming seawater was first pre-filtered with 100-μm-pore filters, then filtered with 10-μm-pore cartridge filters. The filtered seawater was then chlorinated with sodium hypochlorite solution to achieve 20 mg/L of active chlorine concentration and neutralized with sodium thiosulfate after 24 h of chlorination. Prior to stocking shrimp, the rearing water had been fertilized for a week using 2.3 mg L^−1^ urea, 0.1 mg L^−1^ phosphoric acid, and 1.5 mg L^−1^ sodium silicate and was inoculated with cultured diatoms, including 10^4^ cells ml ^−1^ of *Chaetoceros* spp.

### Nursery culture and stocking juveniles

Pathogen-free *L. vannamei* postlarvae were purchased from SyAqua Sia. Co., Ltd. (Thailand). Nursery culture of the postlarvae (mean body weight 0.0015 g) was performed in two separate circular tanks (28 m^2^, 13,214 individuals/m^2^) for 16 days. The postlarvae were fed newly hatched *Artemia nauplii* and extruder pellet (EP) diet (crude protein 35%; CJ feed company, Korea) four times daily. During the culture trial, molasses was provided to each tank as an additional carbon source to adjust the C/N ratio to 15, and groundwater was added to offset water losses due to evaporation. Then, a total of 120,000 nursery-cultured juveniles (initial body weight 0.038 g) were stocked in each raceway tank with a stocking density of 320 individuals/m^3^.

### Feed management and rearing water sampling

Juveniles were fed with newly hatched *A. nauplii* for the first week of raceway culture. Thereafter, *A. nauplii* were replaced by crumble feed and EP diet (crude protein 30%) was provided throughout the experiment until harvest. The feed was supplemented manually three times per day. Daily ratios for the first month were based on 10%–20% of the total shrimp biomass estimated in each raceway. Ratios from the second month were adjusted based on feed consumption, gut fullness, shrimp mean weights, estimated survival, and FCR. Shrimp samples were randomly collected and weighed each week to determine the weekly growth rate (WGR) in each raceway. Feed consumption was monitored prior to each feeding by scooping the bottom of the raceway with a dip net.

To better understand the changes in water quality and bacterioplankton community composition across culture stages, rearing water from both Tank-1 and -2 was sampled for 5 months (152 days) at weekly intervals (Supplementary Tables S1, S2). Thus, sampling was performed on a total of 20 occasions, in which rearing water was sampled at three random locations each time.

### Physiochemical parameters of rearing water

Physicochemical parameters of rearing water *viz.*, temperature, salinity, and pH were measured on-site using a YSI85 multi-parameter instrument (YSI Inc., Yellow Springs, OH, United States) on a daily basis. Similarly, dissolved oxygen (DO) was monitored using a real-time DO-monitoring system (Dongmun ENT). The contents of total ammonia nitrogen (NH4^+^-N, TAN), nitrite-nitrogen (NO_2_^−^-N), nitrate-nitrogen (NO_3_^−^-N), chlorophyll-a (Chl-*a*), total suspended solids (TSS), and volatile suspended solids (VSS) were measured following standard methods (Baird et al., 2012).

### Bacterial diversity assessment

#### Total and *Vibrio*-specific bacterial community analyses

To delineate how growing culture stages influence the bacterioplankton community, 10 rearing water samples, collected from Tank-1 on days 6, 20, 34, 48, 69, 83, 104, 118, 132, and 146, were chosen. These samples were designated as TAS1, TAS2, TAS3, TAS4, TAS5, TAS6, TAS7, TAS8, TAS9, and TAS10 culture stages, respectively, and were subjected to various diversity assessment methods. Firstly, an enumeration of total bacteria and *Vibrio* spp. was performed. Quantification of total bacterial cells followed the previously suggested direct count method (Porter and Feig, 1980). Briefly, 1 ml aliquots of each water sample were fixed with 2% (v/v) formaldehyde solution and stained with 4′, 6-diamidino-2-phenylindole (DAPI, Sigma-Aldrich, United States) for 10 min. The suspensions were then vacuum-filtered through 0.22-μm-pore (47-mm-diameter) black polycarbonate membrane filters (Advantec, Tokyo, Japan). These filters were mounted on glass slides and total bacterial cells were counted under an epifluorescence microscope (Carl Zeiss, Germany) using methods described in our earlier study (Kim et al., 2014).

For determining the load of *Vibrio* spp., serially diluted samples were spread plated on the thiosulfate-citrate-bile salts-sucrose (TCBS, BD Diagnostics) agar medium, which is a well-established and extensively used selective medium to isolate *Vibrio* spp. from aquatic environments (Bolinches et al., 1988). The culture plates were incubated at 30°C for 48 h and the resultant colonies were enumerated as colony-forming units (CFU).

#### Nucleic acid extraction and 16S rRNA gene pyrosequencing

The selected rearing water samples were further analyzed through high-throughput 16S rRNA gene amplicon sequencing. Here, a 1 L volume of each water sample was filtered through a 0.22-μm polyethersulfone membrane filter (Pall Corp., NY, United States), and the resultant filter membranes were used for nucleic acid extraction using a PowerWater® DNA isolation kit (MoBio Laboratories Inc., CA, United States) in accordance with the manufacturer’s instructions. The quality of extracted DNA was ascertained by running extracted DNA on a 1% agarose gel and DNA was quantified using an ND-2000 spectrophotometer (NanoDrop Technologies, DE, United States). Next, PCR amplification of the V1-V3 hypervariable regions of the bacterial 16S rRNA gene was performed using the universal bacteria-specific primer pair: 27F (5′-GAGTTTGATCMTGGCTCAG-3′) and 519R (5′-WTTACCGCGGCTGCTGG-3′), linked with the unique eight-nucleotide barcode and the 454 FLX titanium adaptor sequences. The prepared libraries were then subjected to a clean-up step and evaluated for size and quality on a Bioanalyzer 2,100 (Agilent, Palo Alto, CA, United States) using a DNA 7500 chip. Finally, pyrosequencing was performed using a 454 GS FLX Titanium platform (Roche-454 Life Sciences, CT, United States) at Chun Lab, Inc. (Seoul, Korea) following standard protocol. The generated pyrosequenced amplicons (pyrotags) were processed further for bacterial diversity analysis.

#### Processing, clustering, and diversity analyses of pyrotags

The bioinformatics workflow used for bacterioplankton diversity analysis follows the standard protocol detailed in previous studies (Rajeev et al., 2019, 2021). Briefly, the quality of raw pyrotags was assessed using FastQC software (Andrews, 2010) and a standard quality control criterion was applied to discard low-quality sequences. In this step, we removed all adapter sequences, barcode sequences, short-length reads (<200 nucleotides), and base calls having an average Phred quality score of <30 using the Cutadapt program (Martin, 2011). The retained high-quality reads were then processed in the Quantitative Insights into Microbial Ecology (QIIME, version 1.9.0) pipeline (Caporaso et al., 2010). In QIIME, the reads were first clustered into operational taxonomic units (OTUs) using the classical open-reference OTU picking approach with the UCLUST algorithm (version 1.2.22). The representative OTUs were then assigned taxonomies using the Greengenes reference database (release 13_8; DeSantis et al., 2006) at a 97% similarity threshold. Following this step, we removed all remaining OTUs that merely contained a single sequence (singletons) using the filter_otus_from_otu_table.py script. Moreover, because the present study involves comparative microbiome analysis, the influence of uneven sequencing depth was eliminated by randomly rarifying the whole dataset to the lowest number of reads.

The rarified OTU table was finally used in the QIIME environment to determine alpha diversity indices (Shannon, Simpson’s, and Chao1) and beta diversity indices (Bray-Curtis and weighted UniFrac) and to classify OTUs at various taxonomic ranks.

#### Graphical visualization and statistical analyses

All the graphical visualizations, unless otherwise specified, were made in R (version 4.0.5). To determine the overall bacterial community variation among samples, beta diversity using both non-phylogenetic (Bray-Curtis) and phylogenetic (weighted UniFrac; Lozupone and Knight, 2005) distance metrics were determined, and their clustering pattern was visualized with a principal coordinate analysis (PCoA). To test whether group-specific compositional variations in the bacterioplankton community were statistically significant, an analysis of similarity (ANOSIM, 999 permutations) was conducted in QIIME. Similarly, the linear discriminant analysis (LDA) effect size (LEfSe) method (Segata et al., 2011) was employed to determine discriminatory bacterioplankton communities that varied across different culture stages. The LEfSe analysis was conducted using the online Galaxy interface^1^ with default settings (alpha Kruskal–Wallis value, 0.05; LDA threshold score [log10], 3.0) and differentially abundant bacterioplankton groups were represented using histogram and cladogram. The association between dominant bacterioplankton groups and environmental variables was determined and visualized with a canonical correspondence analysis (CCA).

#### Submission of raw pyrotags in a public repository

The pyrosequencing reads of 16S rRNA gene amplicons supporting the findings of this study were deposited in NCBI’s Sequence Read Archive (SRA) under accession numbers SRR20075654–SRR20075663 and BioProject PRJNA857547.

## Results

### Growth performance and production of shrimp

Results of the *L. vannamei* culture are summarized in Table 1. Beginning when the juveniles were stocked, the bodyweight of shrimp was measured every 2 weeks for 152 days. At the end of the culture, the shrimp’s bodyweight was 15.2 and 13.6 g/individual in Tank-1 and -2, respectively. Total production constituted 1,640 and 1,210 kg, referring to 4.4 and 3.2 kg/m^3^ of productive yield and 287 and 237 individual/m^3^ of the final density. Considering the initial shrimp count, survival rates in the designed BFT systems were estimated to be 89.9% and 73.4% in Tank-1 and -2, respectively. The FCRs ranged from 1.2 to 1.3 in both tanks.

**Table 1:** Summary of *Litopenaeus vannamei* aquaculture reared in BFT system for 152  days.

	Initial BW (g)	Final BW (g)	Stocking (shrimps/m^3^)	Yield (kg/m^3^)	Total production (kg)	Survival rate (%)	FCR
Tank-1	0.038	15.2	320	4.4	1,640	89	1.2
Tank-2	0.038	13.6	320	3.2	1,210	74	1.3

BW, body weight; FCR, feed conversion rate.

### Rearing water quality changes across the culture stages

**Figure 1:**
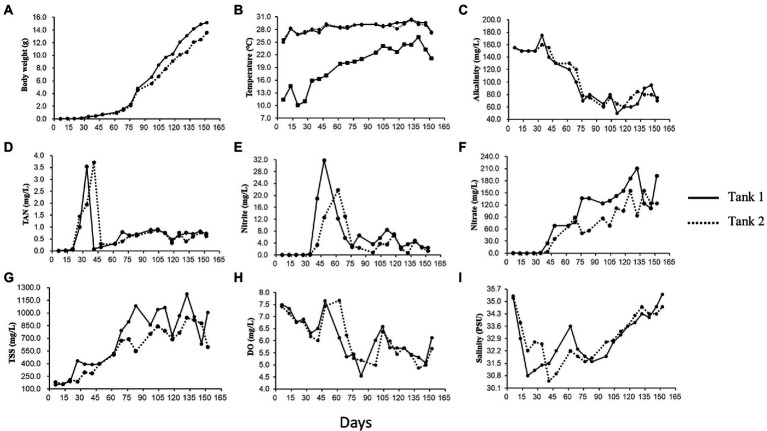
Body weight of the reared *Litopenaeus vannamei* and physicochemical parameters of rearing water over 152 growth days in a biofloc aquaculture system. Curves show values of **(A)** body weight, **(B)** temperature of rearing water (upper curves) and air (lower curves), **(C)** alkalinity, **(D)** total ammonia nitrogen (TAN), **(E)** nitrite (NO_2_-N), **(F)** nitrate (NO_3_-N), **(G)** total suspended solids (TSS), **(H)** dissolved oxygen (DO), and **(I)** salinity in culture Tank-1 and -2.

The sample-specific details on shrimp bodyweight and physicochemical characteristics of rearing water in both tanks are provided in Supplementary Tables S1, S2. Variation in the important physicochemical parameters *viz.*, body weight, temperature, alkalinity, inorganic nutrients (TAN, nitrite, nitrate), TSS, DO, and salinity is represented in Figures 1A–I. As can be seen, the bodyweight of reared shrimp increased gradually over the culture period, rising from 0.38 to 15.2 g. The rearing water temperature was relatively constant, ranging between 25.0–30.4°C in Tank-1 and 25.6°C–30.2°C in Tank-2; however, the air temperature fluctuated considerably, ranging between 10.1°C and 26.2°C. Over the study’s course, the alkalinity gradually declined, from 70 to 155 mg CaCO_3_/L. DO concentrations were maintained over 5.0 mg/l, except for a few days. The content of TAN and nitrate irregularly fluctuated during the study period. For example, the content of TAN initially increased and decreased abruptly. Thereafter, it gradually climbed again and eventually become stable. Similar to TAN, a sudden increase, followed by a decrease in nitrate concentration was observed initially. After, a gradual increase and decrease occurred until the end of the culture. The values of TSS, VSS, and turbidity increased 4, 6, and 12 times, respectively, compared to the initial concentration, with several fluctuations. Finally, Chl-*a* concentration exhibited dramatic changes, ranging from 14–15 to 356–408 μg/l.

### Bacterioplankton diversity and community richness in the rearing water

Total bacterial cell count using DAPI staining showed a fluctuation in the bacterioplankton community over the culture stages (Table 2). Furthermore, we observed a slight upsurge in microbial community abundance along the culture stages; the total bacterial count reached 1.06 × 10^7^ cells ml^−1^ in the culture’s middle (culture stage TAS4) and 1.95 × 10^6^ at the end of the culture (TAS10), as compared to starting count of 9.95 × 10^4^ (TAS1). On the other hand, a *Vibrio*-specific assessment, employing a selective medium (TCBS), found a modest abundance of *Vibrio* spp. throughout the sampling campaign. The *Vibrio* population accounted for 9.0 × 10^1^ to 3.4 × 10^3^ CFU ml^−1^ of rearing water.

**Table 2:** Total bacterial counts, *Vibrio* spp. counts and pyrosequencing output of rearing water.

Growth days (culture stages)	Bacterial counts		Overview of pyrosequencing data					
	Total bacterial count	*Vibrio* count (CFU ml^−1^)	No. of raw reads	No. of qualified reads^*^	No. of OTUs	Shannon	Simpsons	Chao1
6 (TAS1)	9.95 × 10^4^	2.00 × 10^2^	14,218	6,660	596	6.53	0.953	949
20 (TAS2)	5.76 × 10^5^	3.46 × 10^3^	9,054	5,927	452	5.47	0.903	786
34 (TAS3)	3.16 × 10^6^	1.97 × 10^2^	12,099	7,845	614	6.40	0.949	972
48 (TAS4)	1.06 × 10^7^	9.00 × 10^1^	14,326	10,024	739	6.62	0.947	1,485
69 (TAS5)	4.86 × 10^6^	2.40 × 10^2^	7,639	3,545	1,062	9.09	0.994	1,259
83 (TAS6)	9.20 × 10^5^	3.30 × 10^2^	11,169	7,771	606	7.11	0.968	1,053
104 (TAS7)	5.25 × 10^6^	7.50 × 10^2^	14,395	10,329	573	6.14	0.931	1,130
118 (TAS8)	1.15 × 10^7^	2.60 × 10^3^	14,320	10,929	534	6.74	0.969	1,076
132 (TAS9)	3.98 × 10^6^	3.00 × 10^2^	8,729	5,963	651	6.83	0.958	1,307
146 (TAS10)	1.95 × 10^6^	5.13 × 10^2^	12,800	8,857	442	5.64	0.917	819

The alpha diversity indices (Shannon, Simpsons, and Chao1) were calculated at 3% sequence divergence. OTUs, Operational taxonomic units; CFU, Colony-forming units.

* Pyrosequencing reads that passed the quality control criteria.

The details of the pyrosequencing effort employed in this study are provided in Table 2. The pyrosequencing run obtained a total of 118,749 raw sequences. After applying quality control criteria, a total of 77,850 high-quality pyrotags (ranging from 3,545 to 10,929) were retained for diversity analyses. The clustering of these high-quality pyrotags at a 97% similarity threshold resulted in a total of 3,918 OTUs with an average of 626 OTUs per sample (ranging from 442 to 1,062). Initially, we plotted the numbers of high-quality sequences against the Chao1 diversity index as a rarefaction curve (Supplementary Figure S3). The Chao1 curves of each analyzed sample grew with increasing sequencing depth and finally reached a saturation stage, indicating that the number of high-quality pyrotags obtained in our study appeared technically adequate to survey most of the phylogenetic information. After random subsampling (rarefaction) of pyrotags in each sample to the shallowest number of sequences (3,545; TAS5), alpha diversity indices were calculated. These indices were estimated to range from 5.47 to 9.09 for Shannon, 0.903 to 0.969 for Simpson’s, and 786 to 1,485 for Chao1 (Table 2). Though there was no discernible pattern of variation in these indices, their values changed considerably along with the culture stages, with the highest increase during TAS5.

### Bacterioplankton community composition in rearing water alters across the culture stages

**Figure 2:**
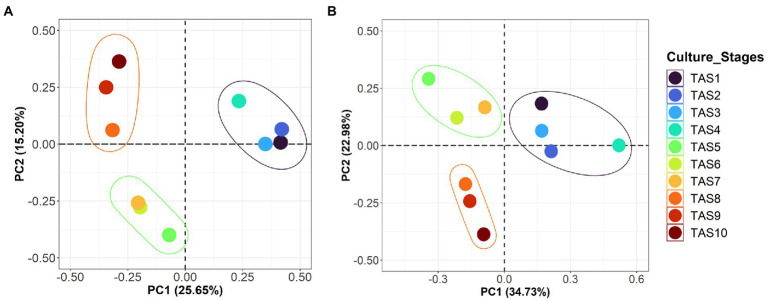
Compositional variations of bacterioplankton community in rearing water. Principal coordinate analysis (PCoA) plots represent beta diversity variations based on Bray-Curtis **(A)** weighted UniFrac **(B)** distance metrics in rearing water samples collected across various culture stages from a commercial BFT-based aquaculture system. The designated sample IDs (TAS1 to TAS10) represent shrimp *Litopenaeus vannamei* culture stages in terms of their growth days 6, 20, 34, 48, 69, 83, 104, 118, 132, and 146, respectively, in culture Tank-1.

To evaluate the similarity/dissimilarity pattern of bacterioplankton communities along the culture stages, the whole community was compared using beta diversity indices Bray-Curtis and weighted UniFrac (Figures 2A,B). The clustering pattern of these indices in PCoA plots identified a strong influence of the culture stage on rearing water microbiota. Therefore, all samples were grouped into three distinct clusters on both the Bray-Curtis-based PCoA (variation explained by PC1 = 25.6%; PC2 = 15.2%) and the weighted UniFrac-based PCoA (PC1 = 34.7% and PC2 = 22.9%). The first group comprising TAS1–4 culture stages clustered distinctly from the other two groups, which consisted of culture stages TAS5–7 and TAS8–10. Further analysis using ANOSIM indicated that the bacterioplankton community variation based on Bray–Curtis dissimilarity was statistically significant (R value = 0.608, *p* = 0.002).

**Figure 3:**
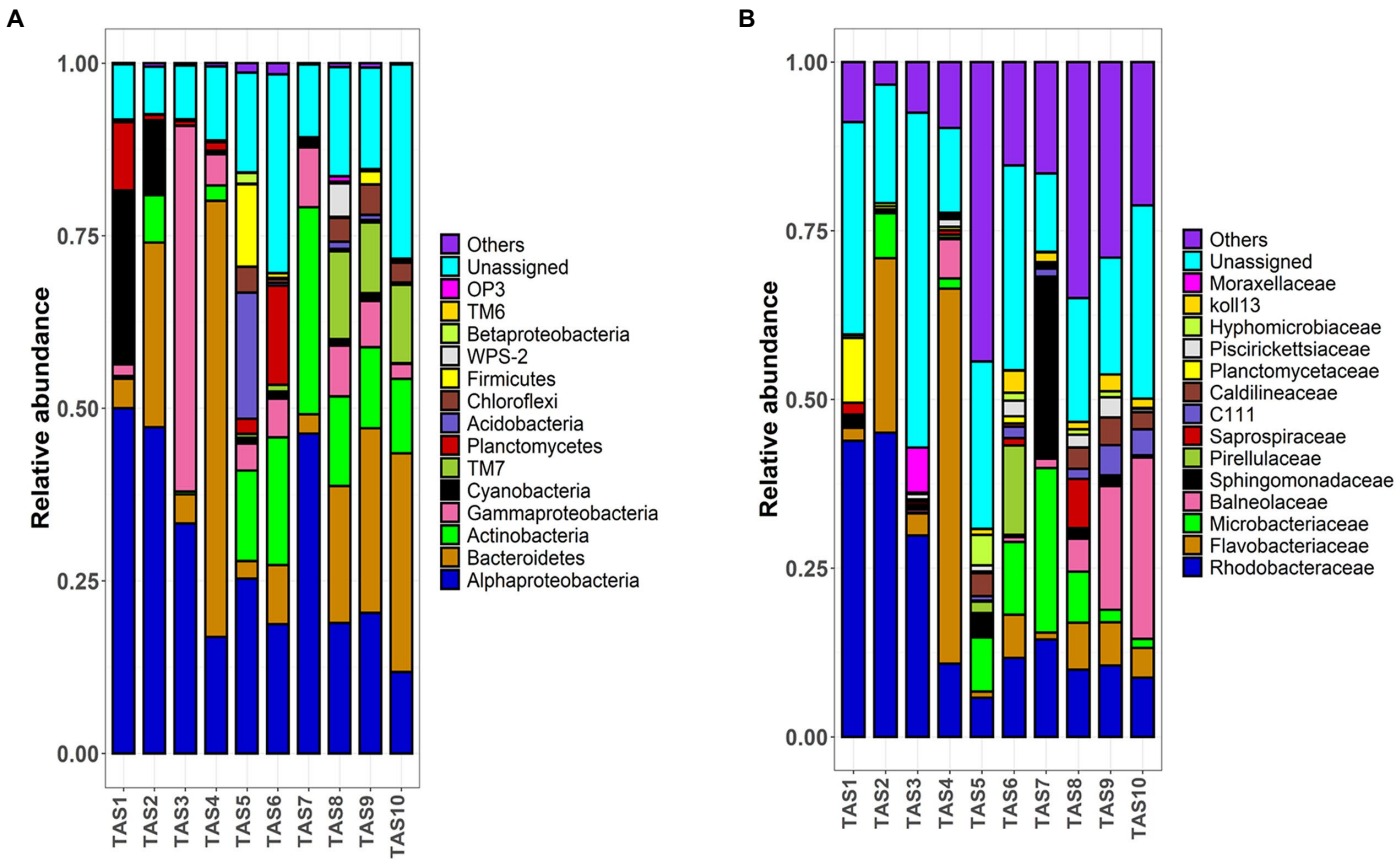
Bacterioplankton community dynamic in rearing water of a biofloc aquaculture system. Stacked bar plots depict the relative abundance of top bacterioplankton taxa at **(A)** phylum/class and **(B)** family levels during various culture stages. The proportions of dominant taxa are only shown. “Others” denotes cumulative relative abundances of remaining taxa. The indicated sample identifiers (TAS1 to TAS10) correspond to different shrimp culture stages in culture Tank-1 based on their growth days 6, 20, 34, 48, 69, 83, 104, 118, 132, and 146, respectively.

The pyrotags were next classified into taxonomic ranks. The relative proportion of the most dominant bacterioplankton taxa at the phylum/class level is shown in Figure 3. The bacterioplankton communities of all culture stages were dominated by similar bacterial groups such as *Alphaproteobacteria*, *Gammaproteobacteria*, *Deltaproteobacteria*, *Bacteroidetes*, *Actinobacteria*, and *Planctomycetes* (Figure 3A). However, their proportions varied considerably among the culture stages. Members of the phylum *Cyanobacteria* dominated the bacterioplankton community in the initial culture stages (25.1% and 10.6% in TAS1 and TAS2, respectively). However, their proportion declined in the later stages (<2% during TAS3–TAS10). In contrast to *Cyanobacteria*, several bacterial groups occupied a minor proportion of the community initially but grew to dominate the community in the later culture stages. For example, *Actinobacteria* dominated the community during culture stages TAS5–TAS7 (13.1%, 18.5%, and 29.9%, respectively) and TAS8–TAS10 (12.9%, 11.7%, and 10.7%, respectively) compared to TAS1–TAS4 (0.3%, 6.8%, 0.4%, and 2.2%, respectively). Similarly, *Chloroflexi* were abundantly present during culture stages TAS5–TAS7 (3.7%, 0.5%, and 0.3%, respectively) and TAS8–TAS10 (3.4%, 4.4%, and 2.8%, respectively) but nearly absent during TAS1–TAS4 (0.0%, 0.0%, 0.0%, and 0.03%, respectively). On the other hand, members of the phylum *TM7* were prominent exclusively at the later culture stages (TAS8–TAS10), while they were present in only a minor proportion initially (TAS1–TAS7). It is important to emphasize that temporal variation in the bacterioplankton community was relatively high at the beginning stages (TAS1–TAS4) but became stable at later culture stages (TAS5–TAS10).

Bacterial community variation along the culture stages was more interesting and visible when taxonomic consideration extended to the family level (Figure 3B). As observed previously, each culture stage was dominated by specific bacterioplankton taxa. For example, *Rhodobacteraceae* represented the most dominant bacterial family (>29%) in culture stages TAS1–TAS3, but it lost that dominance in later culture stages (TAS4–TAS10). In contrast to *Rhodobacteraceae*, members of *Microbacteriaceae* appeared to dominate during TAS5–TAS10, but were absent during TAS1–TAS4 (except for TAS2). Similarly, members of *Balneolaceae* were prominent (>4%) during the later culture stages (TAS7–TAS10) but made up a minor fraction of the community in other stages.

**Figure 4:**
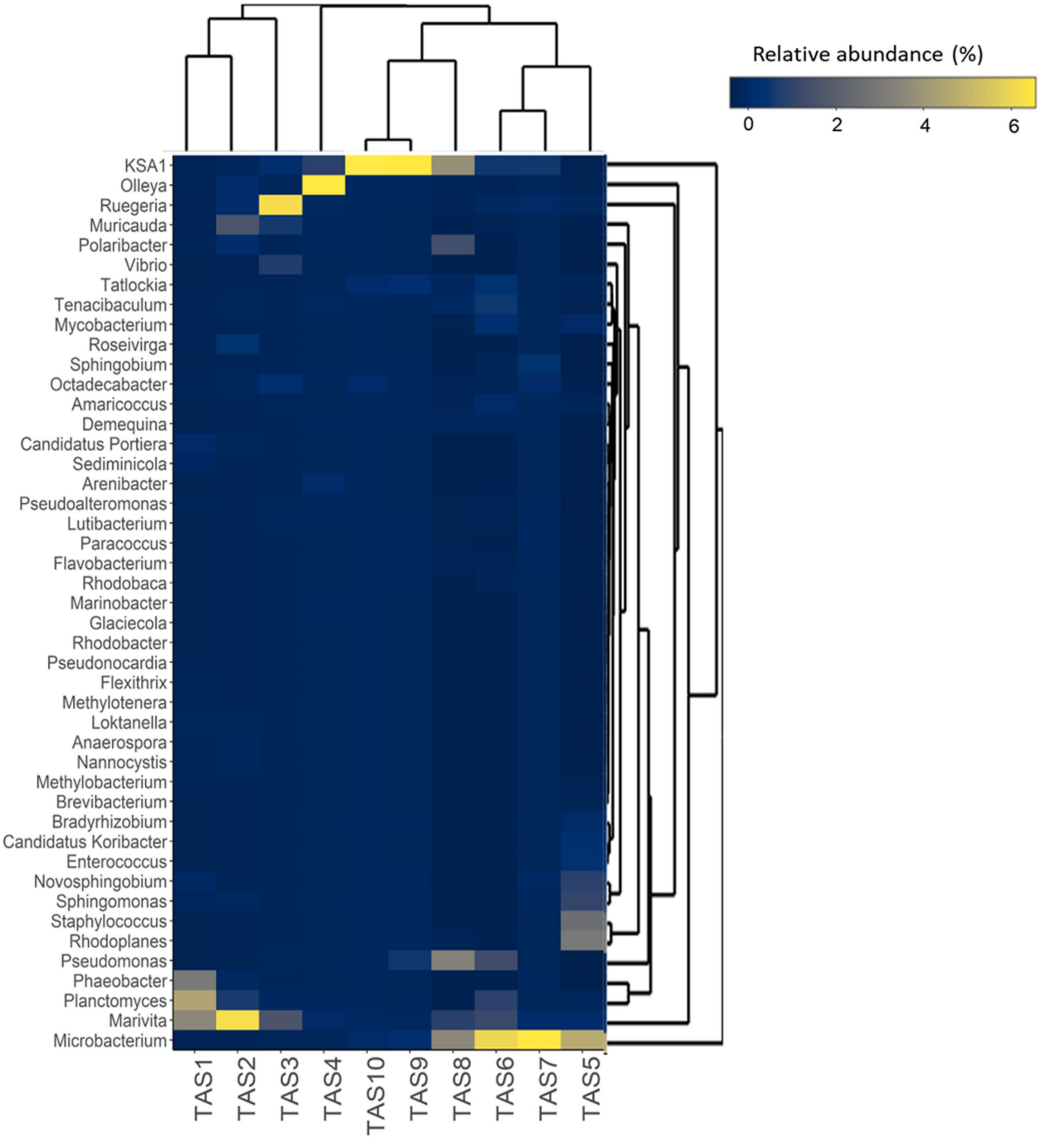
Temporal dynamics in bacterioplankton community composition of rearing water at the genus level. The hierarchically clustered heatmap shows the relative abundance of dominant (top 45) bacterioplankton genera in the rearing water during various culture stages. The color intensity in each cell represents the relative abundance of bacterial genera (listed to the left of the figure) at various shrimp culture stages (indicated at the bottom of the figure), according to the color scale at the right. The culture stages (TAS1 to TAS10) represented here denote shrimp *Litopenaeus vannamei* growth days 6, 20, 34, 48, 69, 83, 104, 118, 132, and 146, respectively.

To decipher the impact of culture stages at lower taxonomic ranks, pyrotags were also analyzed at the genus level and the relative abundances of the most abundant genera were visualized in a heatmap (Figure 4). As can be clearly deduced from the hierarchical clustering pattern, several dominant genera exhibited dramatic changes with the culture and therefore investigated culture stages were distinctly grouped into three large clusters. The first cluster, comprising TAS1–4 culture stages (though TAS4 grouped weakly), was dominated by *Planctomyces*, *Marivita*, *Phaeobacter*, *Ruegeria*, *Olleya*, *Octadecabacter*, and *Muricauda*. The bacterial genera *Planctomyces*, *Marivita*, *Novosphingobium*, *Microbacterium*, *Sediminicola*, *Rhodoplanes*, and *Staphylococcus* were predominant in the second cluster (culture stages TAS5–7) and the genera *Polaribacter*, *KSA1*, *Pseudomonas*, and *Microbacterium* dominated in the third cluster (culture stages TAS8–10).

### Bacterioplankton community dynamics over the culture stages are marked by specific bacterioplankton taxa

**Figure 5:**
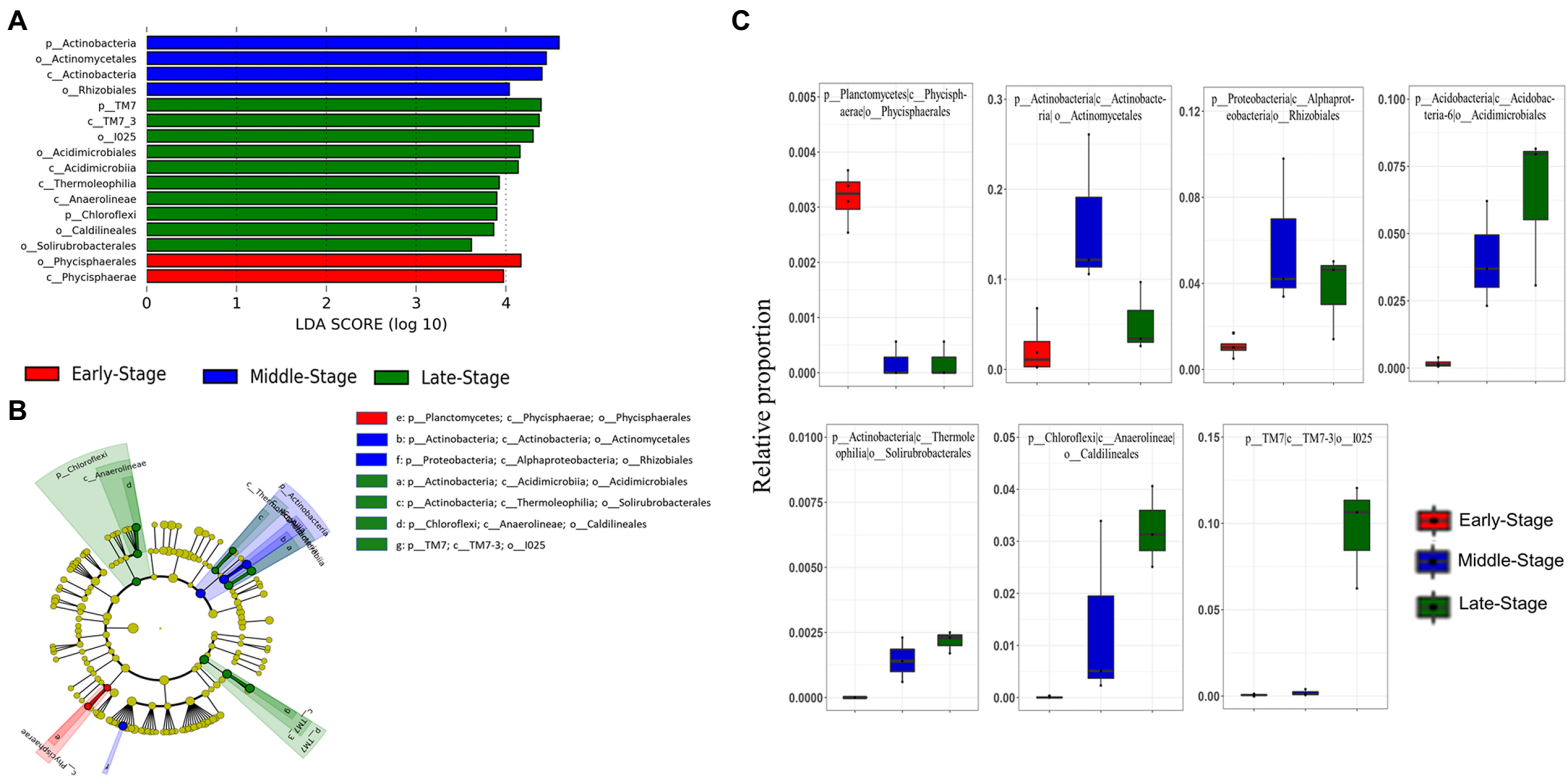
Linear discriminant analysis (LDA) effect size (LEfSe) shows the most differentially abundant bacterioplankton groups as a histogram **(A)** and cladogram **(B)** in rearing water of a BFT-based aquaculture system during various culture stages. The relative proportion of the identified bacterioplankton taxa through LEfSe are represented as boxplots **(C)**. Bacterioplankton groups that possess an LDA threshold > 3 are only represented. In the cladogram, each ring indicates a taxonomic level (e.g., center to outermost as phylum, class, order), while each circle indicates a bacterioplankton group. The taxonomic groups highlighted with different colors signify their enrichment in a particular culture stage.

Considering the clear dynamics of the microbiota composition of rearing water, we next sought to identify differentially abundant bacterioplankton taxa during various culture stages using LEfSe. To do so, all of the investigated samples were broadly classified into three groups; the first group (termed “early-stage”) includes samples TAS1–TAS4 (*n* = 4), while the second group (termed “middle-stage”) includes TAS5–TAS7 (*n* = 3), and the third group (termed “late-stage”) includes TAS7–TAS10 (*n* = 3). The sample grouping strategy adopted here is based on the similarity/dissimilarity patterns observed in the PCoA and heatmap (Figures 2, 4). The obtained LEfSe result at phylum to order levels, as illustrated in the cladogram and histogram, identified a number of bacterioplankton group(s) whose abundances were significantly overrepresented (LDA score > 3) in the early-stage, middle-stage, and late-stage of culture (Figures 5A,B). The abundance of phylum *Planctomycetes* (particularly class *Phycisphaerae*, order *Phycisphaerales*) was especially enriched in the early-stage compared to the other two culture stages. Similarly, the differentially abundant bacterioplankton taxa in the middle-stage belonged to the phyla *Actinobacteria* (class *Actinobacteria*, order *Actinomycetales*) and *Proteobacteria* (class *Alphaproteobacteria*, order *Rhizobiales*), while the late-stage was associated with enrichment of the phyla *Actinobacteria* (classes: *Acidimicrobiia*, and *Thermoleophilia*, and orders: *Acidimicrobiales*, and *Solirubrobacterales*), *Chloroflexi* (class *Anaerolineae*, order *Caldilineales*), and *TM7* (class *TM7*-3, order *I025*). The relative proportions of the identified biomarker taxa (orders) in the categorized culture stages are also represented as box plots (Figure 5C).

### Physicochemical parameters responsible for bacterioplankton community variation

**Figure 6:**
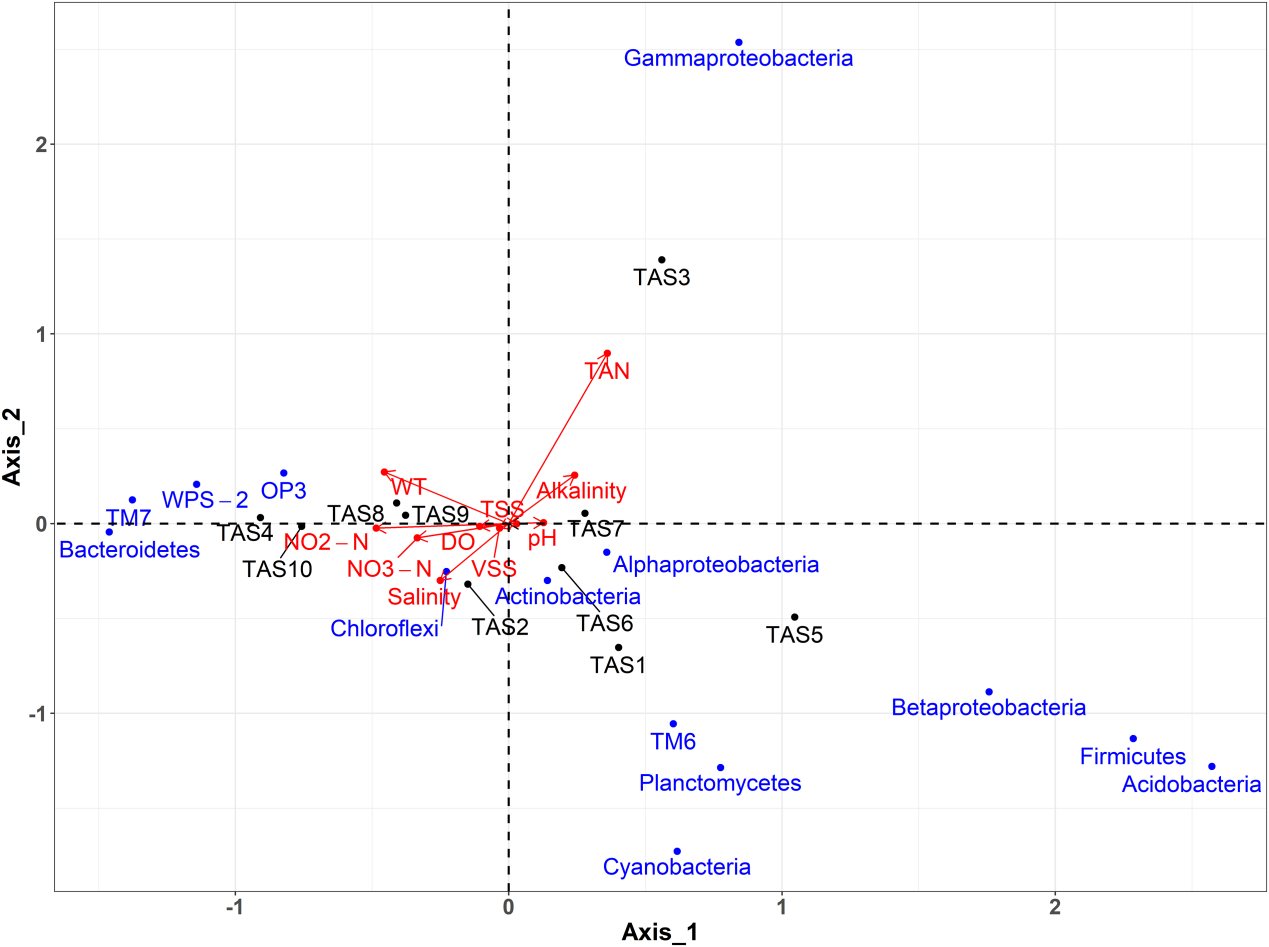
Physicochemical parameters of rearing water responsible for bacterioplankton community changes. Canonical correspondence analysis (CCA) shows an association between the dominant bacterioplankton groups (at phylum/class level) and the physicochemical properties of rearing water. WT, water temperature; TAN, Total ammonia nitrogen; TSS, total suspended solid; VSS, volatile suspended solid. The culture stages (TAS1 to TAS10) represent shrimp *Litopenaeus vannamei* growth days 6, 20, 34, 48, 69, 83, 104, 118, 132, and 146, respectively.

To further enhance our understanding of the physicochemical parameters that could contribute to observed bacterioplankton community variation in rearing water, a CCA was performed (Figure 6). Results of the CCA showed a culture stage-specific distribution of the physicochemical characteristics of rearing water. A positive correlation existed between salinity, nitrite, nitrate, and DO, whereas a negative correlation existed between these variables and TAN and alkalinity. Concerning the bacterioplankton communities, the class *Gammaproteobacteria* was found to be influenced by TAN and alkalinity. Likewise, temperature, nitrate, nitrate, DO, and salinity jointly structured members of *Chloroflexi*, *Bacteroidetes*, *TM7*, *WPS*-2, and *OP3*. Contrary to these bacterioplankton groups, members of *Alphaproteobacteria*, *Actinobacteria*, *TM6*, *Planctomycetes*, *Cyanobacteria*, *Betaproteobacteria*, *Firmicutes*, and *Acidobacteria*, experienced a negligible impact from any of the investigated physicochemical parameters.

## Discussion

Successful implementation of the BFT aquaculture system necessitates the growth of definite microbial communities that are capable of improving water quality, productivity, and biosecurity. There is now mounting evidence that the rearing water microbiota in shrimp aquaculture systems is closely associated with shrimp intestinal microbiota and rearing water microbiota has also been linked to shrimp disease occurrence (Xiong et al., 2018). However, studies remain inconclusive about the bacterioplankton community composition of rearing water and how it changes with shrimp growth.

In the present study, we aimed to understand the changes in physicochemical parameters and bacterioplankton community composition of rearing water during various growth stages of *L. vannamei* in a BFT-based aquaculture system. To promote biofloc formation, we employed a C/N ratio of 15, which is suggested to be the appropriate ratio as compared to other ratios such as 5 and 10 (Panigrahi et al., 2019). Our BFT system yielded an average of 3.6 kg/m^3^ *L. vannamei*, matching the 3–6 kg/m^3^ *L. vannamei* yield recovered in previous BFT systems (Samocha et al., 2007; Krummenauer et al., 2011). This yield is also substantially higher than the 0.2–0.3 kg/m^3^ *L. vannamei* recovered from traditional flow-through culturing systems (Sowers et al., 2006; Krummenauer et al., 2010). Similarly, survival rates (89% and 74% in Tank-1 and -2, respectively) in our systems are within the range of those obtained from other BFT (Samocha et al., 2007; Krummenauer et al., 2011) and traditional flow-through systems (Sowers et al., 2006; Krummenauer et al., 2010). In our study, the poor survival rate (74% in Tank-2) might be explained by shrimp densities, which were higher than the super-intensive density (300 individual/m^3^; Ray, 2012). The FCRs were recorded between 1.2 and 1.3, which are similar to those previously reported in a BFT system (Samocha et al., 2007), but considerably lower than those of the traditional flow-through systems (1.9–2.4; Sowers et al., 2006; Krummenauer et al., 2010). Considering the above evidence, the BFT systems designed in this study could be considered successful for *L. vannamei* culture.

Physicochemical parameters have been shown to influence the overall performance of BFT systems. DO is one of the crucial parameters affecting the growth and health of rearing animals. Though DO content decreased as culture stages progressed, values always exceeded the desired requirement for shrimp health of 3.0 ppm (Seidman and Lawrence, 1985). The temperature ranged between 25°C and 30°C, which is generally considered ideal for rapid shrimp growth (Wyban et al., 1995). A study revealed that a pH > 7 may exert a negative influence on *L. vannamei* growth (Wasielesky et al., 2006). Although the pH was greater than 7 after 83 days of rearing, *L. vannamei* sustained a constant increase in growth, suggesting its resistance to a wider pH range. Nitrogenous constituents including TAN, nitrite, and nitrate determine rearing water quality and health of rearing animals, and hence their control has received great attention in aquacultural industries (Boardman et al., 2004). The safe levels of TAN, nitrite, and nitrate, for the healthy *L. vannamei* culture, have been identified as 4.0, 25.7, and 127.6 mg/l, respectively (Lin and Chen, 2003). In our study, TAN and nitrite concentrations were mostly maintained below the recommended levels, whereas the nitrate concentration exceeded its safe level in the last few stages. It seems that the nitrate concentration range that could exert a lethal impact on shrimp health is wide and thus a concentration of even 435 mg/l nitrates did not exert a significant detrimental influence on the survival and growth of *L. vannamei* (Kuhn et al., 2010).

In a BFT system, the two principal nitrogen conversion processes are nitrification and assimilation. Nitrification involves sequential oxidation of ammonia to nitrate *via* nitrite by chemoautotrophic nitrifying bacteria, whereas assimilation involves direct conversion of ammonia to biomass by heterotrophic bacteria (Ebeling et al., 2006). In our BFT system, as expected, a sudden drop of ammonia was observed, which supports the preferred assimilation process by heterotrophic bacteria. However, an equivalent alteration of nitrite and gradually increasing concentration of nitrate implies the existence of a nitrification process. Parallel to our observation, studies have found high levels of nitrate build-up in the BFT system (Nootong et al., 2011), which may be ascribed to heterotrophic nitrification. Recent studies have demonstrated that the nitrification process, to some extent, is also carried out by heterotrophic nitrifiers (Stein, 2011; Rout et al., 2017).

Microbial communities of rearing water in a BFT system are involved in several crucial functions including biofloc formation, maintenance of water quality and shrimp health, and biogeochemical phenomena (Huang et al., 2020). Moreover, several studies investigating the microbiome composition of aquaculture systems have identified a tight link between the microbiomes of shrimp intestines and rearing water (Zheng et al., 2017; Xiong et al., 2018). The speculated association was explained by the constant flow of rearing water through the digestive tract of growing shrimp, which thus influences the composition of gut microflora (Gatesoupe, 1999).

In the present study, we first demonstrated that the bacterioplankton community in rearing water is highly dynamic. A clear change in overall bacterioplankton community composition along the culture growth was observed. The bacterioplankton community exhibited two major shifts during the entire course of the study. The first shift likely occurred between TAS4 and TAS5 (during 48–69 culture days), to which an abruptly increased abundance of *Actinobacteria* might have contributed majorly. Similarly, the second shift began between TAS7 and TAS8 (during 104–118 culture days), which may be partially attributed to the increased proportion of *Chloroflexi* and *TM7* (a candidate phylum, also called *Saccharibacteria*). Similar patterns in the bacterial community of rearing water, along with intestine and sediment, at different culture stages were previously observed in a shrimp aquamimicry system (Zeng et al., 2020).

Further analysis revealed a complex microbial community in rearing water, comprising several bacterioplankton phyla such as *Proteobacteria*, *Actinobacteria*, *Bacteroidetes*, and *Planctomycetes*. *Proteobacteria* was the most dominant phylum throughout the culture stages, which is consistent with several earlier studies (Hou et al., 2017; Zheng et al., 2017). Similarly, *Bacteroidetes* constituted the second most abundant phylum, but showed a wide range of relative abundance, ranging from 2.5% to 63%. This phylum contains some of the dominant bacteria in algal blooms, and is known for degrading macromolecules in aquatic habitats (Buchan et al., 2014). The abundance of some dominant bacterioplankton phyla exhibited an inconsistent pattern during the culture stages. For instance, *Cyanobacteria* dominated the community in the beginning stages, but then diminished in the later culture stages. One possible explanation for such an unusual pattern is that most *Cyanobacteria* are free-floating photosynthetic autotrophs, and the dense turbidity of bioflocs in rearing water might have hampered their photosynthetic efficiency (Kim et al., 2021). In contrast to *Cyanobacteria*, the phylum *Actinobacteria* showed an entirely opposite trend by totally disappearing in the early culture stages. At the class level, rearing water microbiota was predominantly characterized by *Alphaproteobacteria*, *Gammaproteobacteria*, and *Bacteroidetes*. The higher occurrence of these bacterioplankton groups in a BFT system is not surprising considering their principal characteristics, including the requirement of organic material and nitrogen for growth (Cardona et al., 2016).

At the finer taxonomic level, we observed the predominant existence of the family *Rhodobacteraceae* throughout the investigation, which agrees with the findings of an earlier study (Cardona et al., 2016). The observed higher dominance of *Rhodobacteraceae* members might be attributed to carbon supplementation, a key characteristic of BFT systems. A recent study by Huang et al. (2022) observed that the addition of carbohydrates (e.g., glucose) facilitates *Rhodobacteraceae* growth in BFT systems. In our study, the abundance of the *Rhodobacteraceae* family was about 3 times higher (>39% mean abundance) during the initial culture stages (TAS1–TAS3) than in the late stages (<11% mean abundance during TAS4–TAS10), suggesting a potential role of this taxon in shrimp establishment and maintenance of shrimp health. Several *Rhodobacteraceae* members are known to synthesize vitamin B12, which may support shrimp growth (Sañudo-Wilhelmy et al., 2014).

Several characteristics of *Rhodobacteraceae* make this group a suitable candidate for probiotic application in aquaculture systems. For instance, certain *Rhodobacteraceae* members (e.g., genus *Ruegeria* and *Phaeobacter*) produce tropodithietic acid, which has wide-spectrum antagonistic potential against pathogenic *Vibrio* species such as *Vibrio anguillarum* and thus could reduce larval mortality (Porsby et al., 2008). In fact, a member (*Phaeobacter inhibens*) of this group has proven to be a safe-to-use probiotic in aquaculture (Sonnenschein et al., 2020). Besides their probiotic potential, several *Rhodobacteraceae* members have been reported to possess putative ammonia monooxygenase enzymes, which might support heterotrophic ammonia removal (Crossman et al., 1997). Another abundant family, *Flavobacteriaceae*, excels at decomposing diverse macromolecules such as cellulose and chitin (Mann et al., 2013), which may be profitable in degrading excess feed and shrimp excreta and thus improving rearing water quality (Williams et al., 2013).

At the genus level, rearing water contained several halophilic heterotrophic nitrifying bacteria, such as *Halomonas*, *Pseudoalteromonas*, and *Pseudomonas*, which are known to oxidize higher ammonia concentrations in saline environments (Chankaew et al., 2017). The abundance of these genera in our study differed from those detected in *L. vannamei* earthen pond water by Sombatjinda et al. (2011), who observed a higher abundance of several bacterial genera such as *Flavobacterium*, *Synechococcus*, and *Burkholderia*, as well as autotrophic nitrifiers (e.g., *Nitrobacter*, *Nitrosomonas*, and *Nitrosospira*). The observed disparity may be due to the difference in salinity, which varied from 2 to 10 PSU in the earlier study to 30–35 PSU in our study. The rearing water of aquaculture systems has often been shown to contain potentially pathogenic bacterial genera such as *Vibrio*, *Aeromonas, Photobacterium, Pseudomonas, Candidatus Bacilloplasma,* and *Flavobacterium* (Hou et al., 2017). Among these genera, the most notable pathogens are *Vibrio* spp. and thus quantifying their presence was prioritized in the present study. The existence of *Vibrio* spp. was limited (a minor abundance) in our investigation, which is consistent with earlier similar studies conducted in BFT aquaculture systems (Hoang et al., 2020). The observed low density of *Vibrio* spp. might be due to the water quality and/or higher prevalence of probiotic bacterioplankton taxa that could have restricted their proliferation. Generally, the dominant bacterioplankton members of BFT systems (e.g., *Proteobacteria* and *Bacteroidetes*) compete for food and niche space, limiting the prevalence of pathogens, including *Vibrio* spp. (Wei et al., 2016).

Several antimicrobial agents including antibiotics are being used to control aquaculture pathogens. However, their extensive and indiscriminate use has given rise to the emergence of antibiotic-resistant bacteria and antibiotic-resistant genes in aquaculture production systems (Huang et al., 2015). Due to the higher risk associated with their use, there is growing awareness and strict regulations around the use of antibiotics (Defoirdt et al., 2011). At the same time, this scenario demands effective and environmentally friendly strategies for sustainable aquaculture development. Currently, because of their involvement in shrimp health, water quality, and protection against pathogens, probiotics are considered promising alternatives to antibiotics (Hoseinifar et al., 2018).

Interestingly, we observed a culture stage-specific fluctuation in the abundance of those bacterioplankton groups that could be vital for the effective performance of a BFT system. For instance, *Actinobacteria*, identified as a biomarker taxon during both the middle and late stages of culture, has been frequently described as playing several crucial roles in aquaculture (van der Heul et al., 2018). *Actinobacteria* are recommended as promising candidates for use as probiotics in aquaculture due to several characteristics, such as their ability to degrade macromolecules (e.g., protein and starch) and combat aquaculture pathogens (Das et al., 2008). Previous studies have shown that feed supplemented with members of *Actinobacteria* (e.g., genus *Microbacterium*), could provide health benefits to shrimp by promoting growth and limiting the occurrence of diseases (Das et al., 2008; Skjermo et al., 2015). Additionally, *Actinobacteria* are considered essential for maintaining gut homeostasis (Binda et al., 2018).

Members of the phylum *Chloroflexi* dominated rearing water microbiota in the late-stage of culture. The proportion of *Chloroflexi* members has also been reported to increase with the growth of a commercial fish, *Lateolabrax maculatus* (Duan et al., 2021), indicating that these bacteria might be beneficial in the utilization of accumulated organic matter. Furthermore, certain members of the phylum *Chloroflexi* (e.g., genus *Nitrolancetus*) are known to possess a nitrite oxidoreductase and thus may contribute to the nitrification process by catalyzing nitrite oxidation (Sorokin et al., 2012). Similarly, another biomarker taxon identified during the middle-stage of culture was *Proteobacteria*, whose abundance decreased with increasing culture stages. *Proteobacteria* is a multifunctional phylum in the biofloc-based aquaculture system that possesses the ability to remove nitrogen and degrade organic matter (Cottrell and Kirchman, 2000). In support of our findings, a prior study by Xu et al. (2021) reported that *Planctomycetes* and *Actinobacteria* were biomarker taxa for indoor aquaculture systems. Overall, the LEfSe analysis showed a stage-specific abundance of several beneficial bacterioplankton groups that contribute either to shrimp health or water quality.

In conclusion, we investigated the physicochemical properties and bacterioplankton community of rearing water in a BFT-based *L. vannamei* aquaculture system. Considering the overall performance, the studied BFT system in this study represented a successful and super-intensive *L. vannamei* culture. Our study uncovered the plausible factors shaping the structural composition of the rearing water microbiome, which may help in maintaining rearing water quality and mitigating the occurrence of bacterial diseases in aquaculture systems. Based on our findings, it is reasonable to postulate that shrimp health and production in a BFT-based aquaculture system are largely reliant on rearing water microbiota, besides the shrimp’s own intestinal microbiota. In future studies, it would certainly be interesting to illustrate microbial functional processes in aquaculture systems using shotgun metagenomic and transcriptomic approaches.

## Data availability statement

The pyrosequencing datasets generated for this study are available in the Sequence Read Archive (SRA) database of the National Center for Biotechnology Information (NCBI) under accession numbers SRR20075654–SRR20075663.

## Author contributions

S-KK, JS, SK, and J-CC conceptualized and designed the study. S-KK, JS, SK, and I-KJ collected the rearing water samples and analyzed the physicochemical parameters. S-KK, JS, and SK prepared nucleic acid samples for pyrosequencing. MR and IK performed bioinformatics analyses and interpreted the results. J-CC and IK supervised the study. MR, IK, and J-CC wrote the manuscript. All authors contributed to the article and approved the submitted version.

## Funding

This study was supported by Mariculture Technology Development Using Biofloc of the National Institute of Fisheries Science (NIFS), Incheon (R2022014); by Korea Institute of Marine Science and Technology Promotion (KIMST) funded from the Ministry of Oceans and Fisheries (PM62830); and by the Mid-Career Research Program through the National Research Foundation (NRF) funded by the Ministry of Sciences and ICT (NRF-2022R1A2C3008502).

## Conflict of interest

The authors declare that the research was conducted in the absence of any commercial or financial relationships that could be construed as a potential conflict of interest.

## Publisher’s note

All claims expressed in this article are solely those of the authors and do not necessarily represent those of their affiliated organizations, or those of the publisher, the editors and the reviewers. Any product that may be evaluated in this article, or claim that may be made by its manufacturer, is not guaranteed or endorsed by the publisher.

## Supplementary material

The Supplementary material for this article can be found online at: https://www.frontiersin.org/articles/10.3389/fmicb.2022.995699/full#supplementary-material

## Footnotes

^1^ http://huttenhower.sph.harvard.edu/galaxy
