# Supplementary material for: Exploring bacterioplankton communities and their temporal dynamics in the rearing water of a biofloc-based shrimp (Litopenaeus vannamei) aquaculture system
Source: Front Microbiol. 2022 Sep 20;13:995699. doi: 10.3389/fmicb.2022.995699 (PMC9531771; doi:10.3389/fmicb.2022.995699)
Supplement: Supplementary file 1 [file Data_Sheet_1.DOCX]

**Supplementary Data**

**Supplementary Table S1. Physiochemical parameters of rearing water in culture Tank-1.**

| **Days** | **Water temp. (◦C)** | **Air**  **temp. (◦C)** | **DO (mg/L)** | **Salinity (ppt)** | **pH** | **Alkalinity (mg/L)** | **TAN (mg/L)** | **NO_2_-N (mg/L)** | **NO_3_-N (mg/L)** | **Chl-a (μg/L)** | **TSS**  **(mg/L)** | **VSS**  **(mg/L)** | **Turbidity** | **BW**  **(g)** |
| --- | --- | --- | --- | --- | --- | --- | --- | --- | --- | --- | --- | --- | --- | --- |
| 6 | 25.0 | 11.4 | 7.50 | 35.2 | 8.32 | 155 | ND | ND | 0.17 | 19.38 | 149.20 | 42.40 | 9.8 | 0.03 |
| 13 | 28.2 | 14.6 | 7.34 | 32.9 | 8.15 | 150 | 0.30 | ND | 0.07 | 54.58 | 162.00 | 49.00 | 16.6 | 0.04 |
| 20 | 26.8 | 10.1 | 6.79 | 30.8 | 8.14 | 150 | 0.05 | ND | 0.16 | 325.60 | 187.60 | 58.80 | 25.7 | 0.05 |
| 27 | 27.2 | 11.0 | 6.89 | 31.1 | 7.99 | 150 | 1.00 | 0.01 | 0.12 | 182.17 | 434.67 | 129.33 | 29.3 | 0.12 |
| 34 | 27.9 | 15.9 | 6.32 | 31.4 | 7.90 | 175 | 3.53 | 0.38 | 0.99 | 48.20 | 395.50 | 111.50 | 25.7 | 0.37 |
| 41 | 27.3 | 16.3 | 6.51 | 31.5 | 7.80 | 140 | 0.08 | 18.95 | 24.80 | 66.57 | 388.80 | 102.00 | 22.1 | 0.44 |
| 48 | 29.0 | 17.1 | 7.65 | 32.2 | 8.00 | 130 | 0.16 | 31.79 | 68.20 | 275.46 | 394.40 | 97.60 | 32.1 | 0.66 |
| 62 | 28.4 | 19.9 | 6.12 | 33.6 | 7.50 | 120 | 0.31 | 12.24 | 68.20 | 408.99 | 522.56 | 205.18 | 43.2 | 1.04 |
| 69 | 28.3 | 20.1 | 5.34 | 32.3 | 7.10 | 100 | 0.79 | 5.66 | 76.88 | 344.46 | 791.22 | 285.43 | 109.5 | 1.60 |
| 76 | 29.1 | 20.4 | 5.45 | 31.9 | 6.60 | 70 | 0.65 | 2.67 | 136.40 | 302.05 | 896.73 | 330.00 | 118.8 | 2.30 |
| 83 | 29.2 | 21.0 | 4.55 | 31.6 | 6.57 | 80 | 0.69 | 6.53 | 136.40 | 362.92 | 1084.76 | 349.81 | 152.0 | 4.80 |
| 97 | 28.7 | 22.5 | 6.02 | 31.9 | 6.65 | 65 | 0.88 | 3.59 | 124.00 | 197.26 | 859.06 | 306.19 | 160.4 | 6.60 |
| 104 | 28.7 | 24.1 | 6.59 | 32.7 | 6.70 | 80 | 0.86 | 5.75 | 130.20 | 177.76 | 1040.95 | 356.67 | 177.8 | 8.50 |
| 111 | 28.7 | 23.5 | 5.72 | 33.1 | 6.23 | 50 | 0.75 | 8.46 | 142.60 | 138.26 | 1064.29 | 369.52 | 241.4 | 9.70 |
| 118 | 29.2 | 22.7 | 5.70 | 33.6 | 6.29 | 60 | 0.47 | 6.58 | 155.00 | 147.00 | 732.00 | 360.50 | 196.5 | 10.20 |
| 125 | 29.6 | 24.5 | 5.70 | 33.8 | 6.39 | 60 | 0.63 | 2.58 | 186.00 | 113.92 | 968.78 | 365.97 | 208.1 | 12.10 |
| 132 | 30.4 | 24.4 | 5.43 | 34.3 | 6.55 | 65 | 0.76 | 3.63 | 210.80 | 374.00 | 1223.89 | 426.67 | 250.9 | 13.10 |
| 139 | 29.6 | 26.2 | 5.32 | 34.1 | 6.76 | 90 | 0.71 | 4.55 | 124.00 | 303.13 | 956.25 | 359.00 | 205.0 | 14.20 |
| 146 | 29.6 | 23.3 | 5.10 | 34.7 | 6.79 | 95 | 0.82 | 2.58 | 111.60 | 310.20 | 633.75 | 256.67 | 148.9 | 14.90 |
| 152 | 27.4 | 21.2 | 6.12 | 35.4 | 6.49 | 70 | 0.72 | 2.48 | 192.20 | 308.01 | 1008.57 | 323.33 | 154.6 | 15.17 |
| ND: Not detected | | | | | | | | | | | | | | |

**Supplementary Table S2. Physiochemical parameters of rearing water in culture Tank-2.**

| **Days** | **Water temp. (°C)** | **Air**  **temp. (°C)** | **DO (mg/L)** | **Salinity (ppt)** | **pH** | **Alkalinity (mg/L)** | **TAN (mg/L)** | **NO_2_-N (mg/L)** | **NO_3_-N (mg/L)** | **Chl-a (μg/L)** | **TSS**  **(mg/L)** | **VSS**  **(mg/L)** | **Turbidity** | **BW(g)** |
| --- | --- | --- | --- | --- | --- | --- | --- | --- | --- | --- | --- | --- | --- | --- |
| 6 | 25.6 | 11.4 | 7.42 | 35.3 | 8.34 | 155 | 0.01 | ND | 0.04 | 14.45 | 181.00 | 41.00 | 11.20 | 0.03 |
| 13 | 28.3 | 14.6 | 7.13 | 33.8 | 8.08 | 150 | 0.10 | ND | 0.02 | 73.14 | 154.80 | 42.00 | 16.55 | 0.04 |
| 20 | 26.9 | 10.1 | 6.77 | 32.2 | 8.25 | 150 | 0.09 | ND | 0.04 | 339.12 | 207.60 | 67.60 | 42.77 | 0.05 |
| 27 | 27.5 | 11.0 | 6.79 | 32.7 | 7.98 | 150 | 1.44 | 0.01 | 0.17 | 284.31 | 189.00 | 72.07 | 46.80 | 0.12 |
| 34 | 28.1 | 15.9 | 6.18 | 32.6 | 8.10 | 160 | 1.94 | 0.05 | 0.12 | 173.18 | 297.68 | 86.90 | 39.31 | 0.38 |
| 41 | 28.0 | 16.3 | 6.00 | 30.5 | 7.80 | 155 | 3.71 | 3.27 | 3.72 | 349.22 | 283.33 | 72.08 | 34.97 | 0.46 |
| 48 | 29.3 | 17.1 | 7.44 | 30.9 | 7.80 | 130 | 0.29 | 12.56 | 35.03 | 290.95 | 396.27 | 125.72 | 28.92 | 0.69 |
| 62 | 28.6 | 19.9 | 7.67 | 32.2 | 7.50 | 130 | 0.29 | 21.85 | 66.34 | 232.67 | 509.21 | 179.35 | 34.19 | 0.90 |
| 69 | 28.7 | 20.1 | 6.23 | 31.9 | 7.20 | 120 | 0.40 | 13.02 | 89.28 | 267.73 | 672.00 | 216.00 | 38.77 | 1.40 |
| 76 | 29.1 | 20.4 | 5.27 | 31.6 | 6.82 | 78 | 0.63 | 2.99 | 49.60 | 298.15 | 690.36 | 211.12 | 90.96 | 2.10 |
| 83 | 29.1 | 21.0 | 5.19 | 31.8 | 6.67 | 75 | 0.64 | 2.44 | 55.80 | 356.48 | 547.17 | 232.33 | 138.10 | 4.50 |
| 97 | 29.3 | 22.5 | 4.99 | 32.7 | 6.57 | 60 | 0.80 | 0.83 | 86.20 | 197.26 | 755.00 | 299.38 | 170.10 | 5.60 |
| 104 | 28.9 | 24.1 | 6.38 | 32.8 | 6.90 | 75 | 0.91 | 3.77 | 68.20 | 161.75 | 841.67 | 281.50 | 156.60 | 6.70 |
| 111 | 29.0 | 23.5 | 5.99 | 33.3 | 6.49 | 65 | 0.76 | 3.50 | 111.60 | 141.59 | 786.67 | 327.08 | 177.80 | 7.90 |
| 118 | 28.2 | 22.7 | 5.45 | 33.6 | 6.55 | 60 | 0.34 | 6.99 | 105.40 | 155.49 | 687.92 | 298.33 | 156.50 | 9.10 |
| 125 | 29.3 | 24.5 | 5.70 | 34.2 | 6.57 | 75 | 0.76 | 2.02 | 155.00 | 253.06 | 762.92 | 308.33 | 170.70 | 10.10 |
| 132 | 30.2 | 24.4 | 5.40 | 34.7 | 6.47 | 85 | 0.40 | 0.64 | 93.00 | 314.95 | 943.75 | 340.00 | 181.20 | 10.50 |
| 139 | 29.2 | 26.2 | 4.88 | 34.3 | 6.54 | 80 | 0.60 | 4.79 | 155.00 | 241.91 | 915.83 | 376.67 | 183.90 | 12.10 |
| 146 | 29.1 | 23.3 | 4.99 | 34.3 | 6.79 | 80 | 0.78 | 2.62 | 124.00 | 134.49 | 878.50 | 315.00 | 227.30 | 12.50 |
| 152 | 27.2 | 21.2 | 5.68 | 34.7 | 6.55 | 75 | 0.62 | 1.38 | 124.00 | 83.12 | 598.40 | 197.60 | 121.20 | 13.60 |
| ND: Not detected | | | | | | | | | | | | | | |


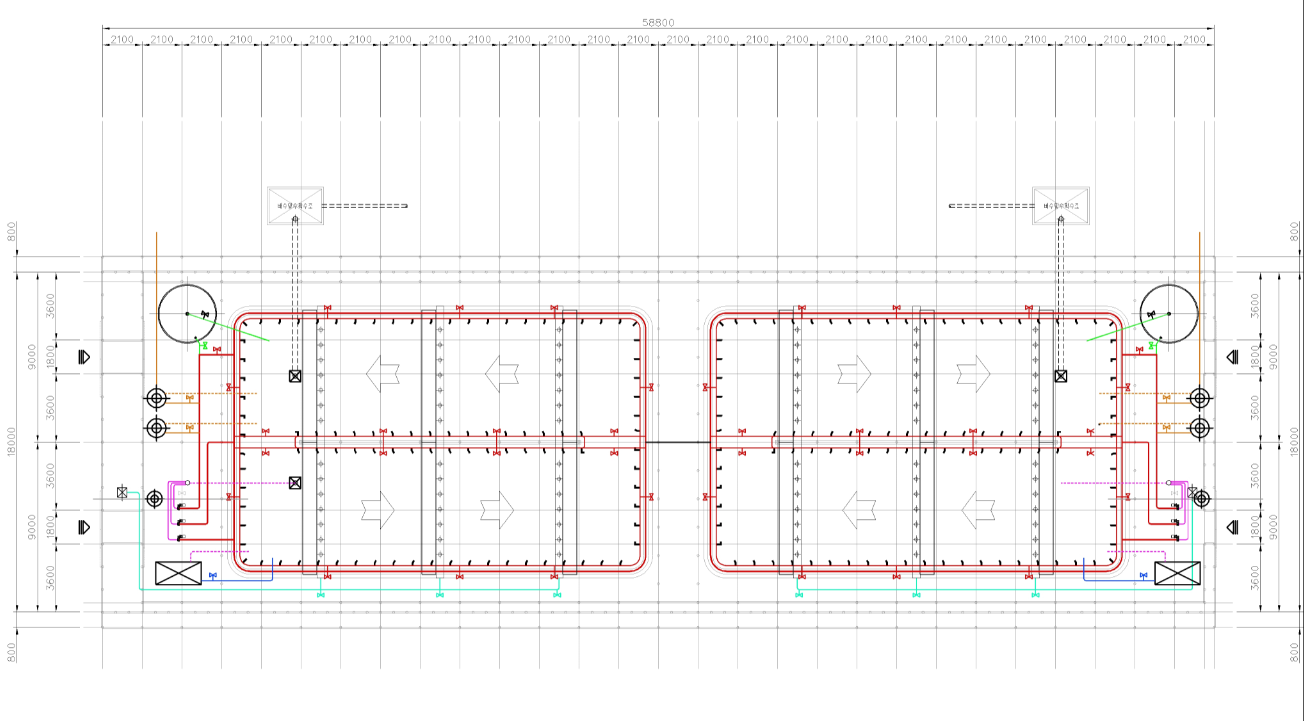


Supplementary Figure S1. The layout of raceway tanks (top view)


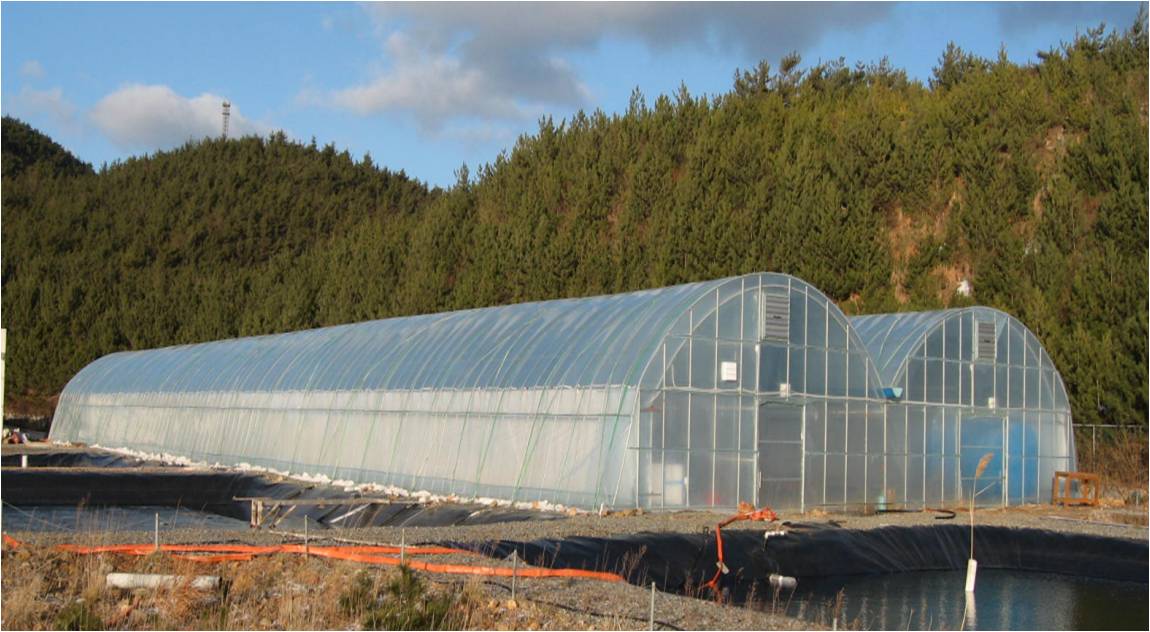


Supplementary Figure S2. A photograph of a greenhouse equipped with raceway tanks.


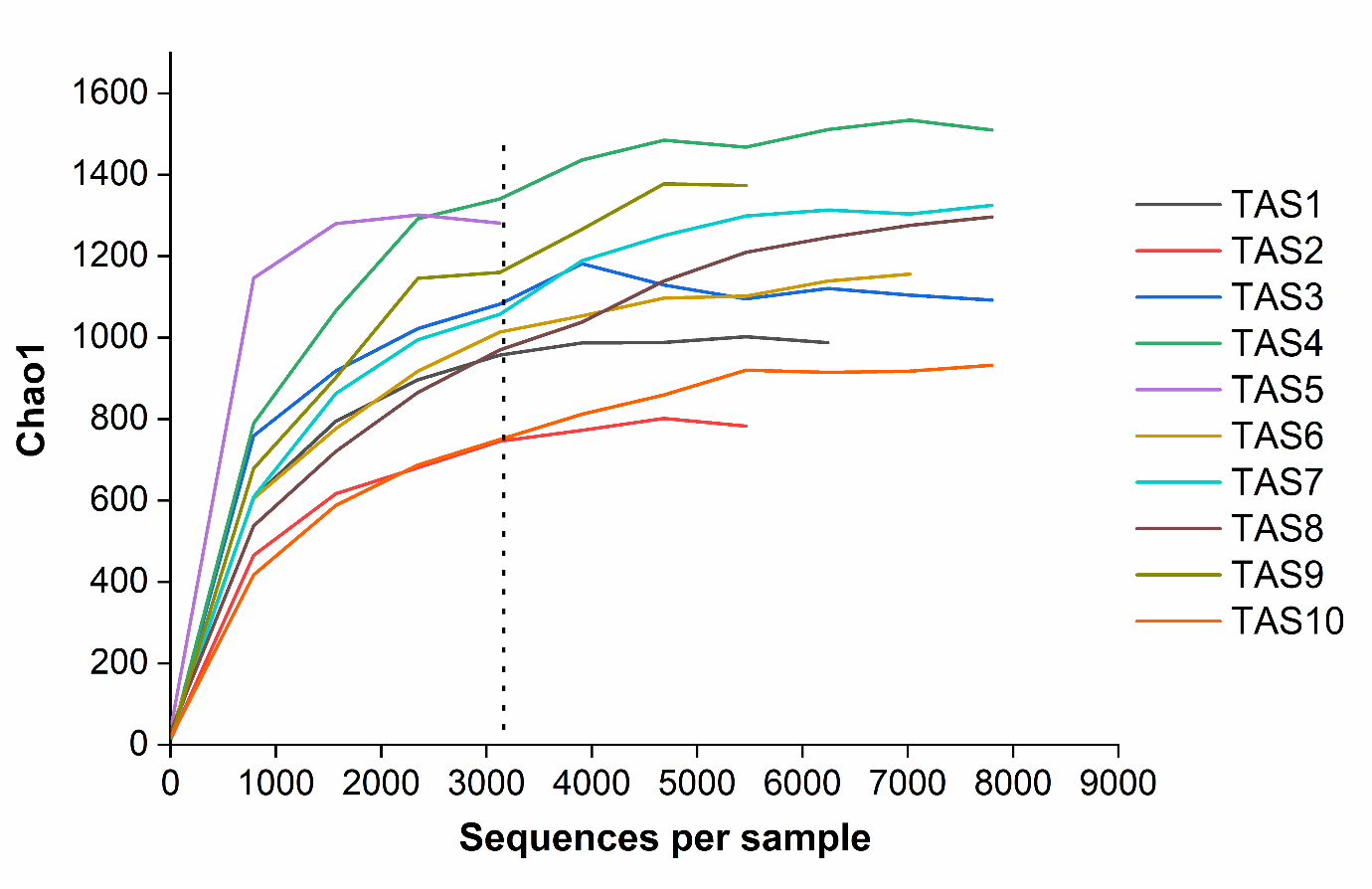


**Supplementary Figure S3.** Rarefaction curves represent the bacterioplankton community of rearing water in a BFT-based aquaculture system. The curves plotted between Chao1 and the number of high-quality pyrotags reached saturation indicating that all samples were sequenced at depths required to evaluate taxonomic features. The vertical line implies the number of sequences (3545) to which the whole dataset was equalized to an identical number.
